# 600 Reconstruction of Finger Contracture with an Expanded Dorsal Metacarpal Artery Perforator Flap

**DOI:** 10.1093/jbcr/irac012.228

**Published:** 2022-03-23

**Authors:** Burak Ozkan, Cagri A Uysal, Ayse Ebru Abali, Mehmet Haberal

**Affiliations:** Baskent University, Ankara, Ankara; Baskent University Faculty of Medicine, Ankara, Ankara; Baskent University, Ankara, Ankara; Baskent University, Ankara, Ankara

## Abstract

**Introduction:**

The first dorsal metacarpal artery perforator (DMCAP) flap is frequently used to cover exposed bone, tendon and neurovascular structures in the hand after trauma and burns. The size and width of DMCAP flap is limited and rotation arc generally lets to cover defects up to middle phalanx. Expansion of the DMCAP flap has not been reported in the literature and this technique might be solution to increase flap viability and size in order to cover defects up to distal phalanx. In this study, we will describe utilization of tissue expander to first DMCAP and present a case of electric burns in with flexor contracture.

**Methods:**

A nine-year-old male patient applied to our clinic with the complaint of inability to extend the second finger of the left hand after an electrical burn. Physical examination revealed flexor contracture in the distal interphalangeal (DIP) and proximal interphalangeal (PIP) joints. Reconstruction was planned for the patient with a two-session expanded first DMCAP flap.

In the first session, a 16 cc 5x3 cm tissue expander placed through a 3 cm vertical incision at the fifth metacarpal level. From the second postoperative week, the tissue expander was inflated with 1 ml of isotonic three days a week. Six weeks later, the DMCAP area was enlarged by giving 45 cc saline.

**Results:**

In the second session, contractures at the level of the left hand 2nd finger DIP and PIP were excised.. Left hand 2nd finger was fixated in extension with K-wire. 9x3 cm DMCAP flap was elevated by dissection over the paratenon and the pedicle was preserved. The flap was adapted to the defect area on the volar face with 180-degree rotation angle. The flap donor site was closed primarily.

There were no complications in the post-operative period. K-wire was removed at 6 weeks postoperatively. The patient was referred to the physical therapy.

**Conclusions:**

In cases where the tissue defect cannot be closed with loco regional flaps, extra tissue can be provided by free flaps or using tissue expanders. Primary closure of the donor area, appropriate flap thickness for the finger, and aesthetically pleasing results are among the advantages of the expanded DMCA flap.. However, in tissue expander applications in the upper extremity, the patient should be followed closely, and pain and finger circulation should be constantly questioned.

